# Advancing Immunotherapy in Pancreatic Cancer

**DOI:** 10.3390/ijms252111560

**Published:** 2024-10-28

**Authors:** Ahmad Hegazi, Lauren Elizabeth Rager, Dean Edward Watkins, Kuo-Hui Su

**Affiliations:** Department of Cell and Cancer Biology, College of Medicine and Life Sciences, The University of Toledo, Toledo, OH 43614, USA; ahmad.hegazi@rockets.utoledo.edu (A.H.); lauren.rager@rockets.utoledo.edu (L.E.R.); dean.watkins@rockets.utoledo.edu (D.E.W.)

**Keywords:** immunotherapy, pancreatic cancer, antibody therapy, vaccine therapy, cellular therapy, clinical trial, immune checkpoint inhibitor

## Abstract

Pancreatic cancer remains one of the deadliest malignancies, with a consistently low five-year survival rate for the past several decades. This is in stark contrast to other cancers, which have seen significant improvement in survival and prognosis due to recent developments in therapeutic modalities. These modest improvements in pancreatic cancer outcomes have primarily resulted from minor advances in cytotoxic chemotherapeutics, with limited progress in other treatment approaches. A major focus of current therapeutic research is the further development of immunomodulatory therapies characterized by antibody-based approaches, cellular therapies, and vaccines. Although initial results utilizing immunotherapy in pancreatic cancer have been mixed, recent clinical trials have demonstrated significant improvements in patient outcomes. In this review, we detail these three approaches to immunomodulation, highlighting their common targets and distinct shortcomings, and we provide a narrative summary of completed and ongoing clinical trials that utilize these approaches to immunomodulation. Within this context, we aim to inform future research efforts by identifying promising areas that warrant further exploration.

## 1. Introduction

Pancreatic ductal adenocarcinoma (PDAC) is the third-leading cause of cancer-related deaths in the United States and the third-leading cause of cancer-related deaths between the ages of 50 and 79 years [1]. Approximately 85% of patients were found to have late-stage unresectable or metastatic disease at diagnosis, resulting in a five-year survival rate of 13%, among the lowest of all cancers [1,2]. Despite rapid advances in the treatment of other solid tumor types with innovative immunotherapies, the prognosis of PDAC has remained dismally poor, with only resectable tumors having curative treatment options in the case of surgery. Currently, the first-line treatment across all pancreatic cancer patients is either a combination regimen with 5-fluorouracil, leucovorin, oxaliplatin and irinotecan (FOLFIRINOX) or liposomal irinotecan (NALIRIFOX), or gemcitabine combined with nab-paclitaxel (GnP) [3,4]. Unfortunately, these modalities are often accompanied by significant toxicities or rendered ineffective by innate or acquired tumor resistance [4]. In the case of cancer progression on first-line therapy, second-line treatment options are available, with immunotherapies only recently being included in this category.

Immunotherapies have emerged as a novel addition to the therapeutic arsenal, which offers new hope for pancreatic cancer treatment. Anti-programmed death protein 1 (anti-PD-1) antibodies, such as pembrolizumab, were the first Food and Drug Administration (FDA)-approved immunotherapeutic agents for PDAC management despite limited benefit. The PD-1/program death ligand-1 (PD-L1) checkpoint allows for tumoral PD-L1 to interact with T-cell PD-1 and prevent T-cell-mediated immune activation; blocking this interaction allows the host immune system to recognize and attack the tumor cells [5]. Although this approach has shown benefits, its usage as second-line therapy is currently limited to patients with mismatch repair deficient (dMMR) tumors because only this subset of cancers has demonstrated a marked response [6]. Mismatch repair deficiencies have been shown to increase mutational accumulation in tumoral cells, enhancing immunotherapy because of an increase of tumor neoantigen production for immune targeting. Unfortunately, mismatch repair deficiency mutations only comprise a small percentage of patients, at approximately 1% [7]. Other mutations are demonstrably more common. 

Most notably, Kirsten rat sarcoma viral oncogene homologue mutants (KRAS) are almost ubiquitous in PDAC, present in approximately 95% of tumors [8]. KRAS is a member of the rat sarcoma (RAS) family of proteins, small guanosine triphosphatases (GTPases) involved in cellular signal transduction. In the wildtype form, KRAS regulates rapidly accelerated fibrosarcoma (RAF), mitogen-activated extracellular signal-regulated kinase (MEK), and extracellular signal-regulated kinase (ERK) as part of cellular growth and proliferation. However, when KRAS is mutated, it often leads to hyperactivation of this signal, making *KRAS* an oncogene that is commonly observed in many solid tumors [9]. Because KRAS is an intracellular protein, it has been challenging to target directly with antibody-based therapies. As a result, antibody-based approaches are prevalent in targeting other components of this pathway in addition to other highly oncogenically active proliferative proteins, such as epidermal growth factor receptor (EGFR) and vascular endothelial growth factor (VEGF) receptor-targeted antibodies. Despite these efforts, such approaches have yet to show significant success in PDAC. Given the limitations of current approaches to targeting these tumor markers, there is growing interest in exploring alternative immunomodulatory approaches that could enable the host immune system to recognize and target these tumor-specific markers. In this review, we will focus on these emerging approaches.

Compared to other solid tumors, PDAC presents unique challenges due to the tumor’s dense fibrotic stroma, low mutational neoantigen burden, and immunosuppressive environment. These characteristics leave immunotherapies unable to effectively penetrate the extracellular matrix and recruit the local adaptive immune system [10,11]. To address these challenges, research has primarily focused on targeting stromal elements to increase tumor vulnerability, identifying multiple common tumor neoantigens for potential drug development alongside the use of adjunct immunomodulatory agents to enhance the efficacy of targeted therapy [12]. Although immunotherapy in pancreatic cancer has historically yielded discouraging results, recent studies using anticancer mRNA vaccines with adjuvant immunotherapy alongside chemotherapy have shown promising results [13]. In this review, we aim to discuss previous unsuccessful attempts to integrate immunotherapy into PDAC treatment and compare them with recent novel interventions that have demonstrated clinical benefit in trials. 

This review of PDAC immunotherapy national clinical trials (NCT) encompasses completed trials between 1996 and 2023 and the ongoing trials initiated in any year. We have categorized the reviewed trials based on the immunomodulatory approach used: antibodies, vaccines, cellular therapies, or a combination of these approaches. Finally, we will discuss ongoing areas of focused investigation essential to increasing the efficacy of immunotherapy in this patient population. 

## 2. Strategy 

### 2.1. Search Strategy

We conducted trial searching on ClinicalTrials.gov, which was initiated in 1996 and ended in 2023. We used the following keywords in different combinations: “pancreatic adenocarcinoma”, “pancreatic cancer”, “pancreatic neoplasm”, “metastatic”, “immunotherapy”, “antibody”, “vaccine”, and “cellular immunotherapy”. We collected results through ClinicalTrials.gov, PubMed, and American Society of Clinical Oncology (ASCO) publications. Two reviewers performed the search process independently and verified the final lists; a third reviewer resolved any discrepancies between them.

### 2.2. Inclusion and Exclusion Criteria

The inclusion criteria for completed trials included: 1—patients diagnosed with pancreatic adenocarcinoma [Stage I, II, III, and IV PDAC patients]; 2—Phase I–III clinical trials; 3—inclusion of immunomodulatory treatment; and 4—inclusion of treatment efficacy in primary or secondary outcomes. We included all active trials on ClinicalTrials.gov in Table 1, irrespective of intended measured outcomes. In addition, we included treatment response and patient survival-based clinical outcomes in Table 2, Table 3, Table 4 and Table 5.

We excluded from Table 2, Table 3, Table 4 and Table 5 completed trials that (1) were terminated with no reason listed; (2) were terminated because of accrual issues; (3) were terminated because of inability to acquire drug or other logistical issues; (4) were completed, but no results were available on ClinicalTrials.gov, published on ASCO, or included in a peer-reviewed publication; or (5) had less than five PDAC patients in the experimental group on conclusion of the trial. We did not exclude any ongoing trials in Table 1, irrespective of patient enrollment. We resolved disagreements between researchers regarding inclusion and exclusion criteria by discussing details and obtaining agreement.

## 3. Mechanisms of Pancreatic Adenocarcinoma Tumorigenesis and Treatment Resistance 

Resistance mechanisms in disease refractory to chemotherapy are primarily driven by tumor heterogeneity via epigenetic changes and random mutations, localized immunosuppression of innate immune cells, and dense tumor stroma [14]. Tumor-associated mesenchymal stem cells, tumor-associated fibroblasts, and tumor-associated myeloid cells facilitate local tumorigenesis through the production of tumor-promoting growth and angiogenic factors, while inhibiting immunomodulatory therapy through the promotion of local immunosuppressive effects using various factors, including transforming growth factor beta (TGF-β), tumor necrosis factor α (TNF-α), and interleukin (IL) 10 [12,15]. Furthermore, mast cell infiltration and reduced expression of major histocompatibility complex (MHC), which are also commonly observed in cases of PDAC, lead to a worse prognosis because of fewer opportunities for tumor-infiltrating T-cell recognition and antitumor effects [12]. These factors contribute to a microenvironment that resists therapeutic interventions, complicating treatment efforts and worsening patient outcomes. 

Another mechanism for the formation of immune-privileged solid tumors is the overexpression of factors involved in extracellular adenosine metabolism, specifically CD39 and CD73. Elevated extracellular adenosine levels due to increased CD39 and CD73 activity have been shown to induce angiogenesis through the enhanced secretion of IL-8 and basic fibroblast growth factor by endothelial cells [16]. This heightened angiogenesis by endothelial cells augments cancer growth of these solid tumors.

High concentrations of extracellular adenosine further promote immune evasion through the inhibition of tumor-infiltrating and tumor-adjacent dendritic cells (DCs), macrophages, neutrophils, natural killer (NK) cells, and CD4+ and CD8+ T cells [17,18,19,20]. PDAC cells have been found to overexpress CD73, exacerbating the role of the tumor microenvironment in promoting treatment failure, weakened immune recognition, and, ultimately, poor outcomes that are regularly observed among patients with pancreatic cancer [21,22,23]. High levels of expression have been significantly correlated with poorer differentiation (*p* = 0.002), larger tumor size (*p* = 0.049), and reduced overall survival (OS) time (*p* < 0.0001) [24]. These significant correlations have led to CD73 becoming a key area of investigation as researchers look to study ways to mitigate its immunosuppressive effects. 

In addition to elevated CD73, tumor cells employ multiple strategies to diminish immune activation, primarily by preventing immune checkpoint recognition. Similar to PD-L1-mediated immune recognition inhibition, other immune checkpoint pathways are exploited, including cytotoxic T-lymphocyte-associated protein 4 (CTLA-4). CTLA-4 is a constitutively expressed protein in regulatory T cells that inhibits cytotoxic T-cell activity through binding to CD80/CD86 on T cells [25]. Within the tumoral microenvironment is a significantly elevated activity of CTLA-4-mediated immune inactivation, leading to minimal immune response to tumor antigens.

Beyond the previously mentioned immune evasion and treatment resistance mechanisms, the majority of pancreatic adenocarcinoma is composed of dense stroma—a conglomeration of fibroblasts, stellate cells, immune cells, extracellular matrix, and vascular components—which acts as a physical barrier to tumor penetration by chemo- and immunotherapeutic agents [11,26,27,28]. This microenvironment is constantly in flux, with compositional changes driving local growth and enforcing the transition from precancerous lesions to malignancy [11,29]. The rigid, fibrous extracellular matrix creates a harsh environment that is hypoxic and nutrient-deprived, leading to abnormal angiogenesis, poor immunogenicity, enhanced tumor progression, and eventual metastasis [26,27,30,31]. Notably, increased local concentrations of TGF-β produced by damaged cells have been shown to induce stellate cell activation and accelerate collagen production, leading to rapid matrix formation [31]. Another key component, mesothelin, shows increasing expression as the tumor progresses into later stages, contributing to tumor growth by promoting proliferation and inhibiting TNF-α-induced apoptosis [32,33,34]. Fibronectin, secreted by pancreatic stellate cells, provides scaffolding in the tumor microenvironment (TME), enhancing proliferative signaling, angiogenesis, and chemoresistance, while downregulating local immunologic activity [35,36]. Although elements of the stroma, particularly hyaluronan and mesothelin, have been identified as targets of adjuvant pharmacotherapies to increase tumor vulnerability to chemo- and immunotherapies, the results thus far have been lackluster [37,38]. Given the numerous barriers to adequate immune cell identification, penetration, and destruction of tumor cells with current treatment modalities, immunomodulatory approaches provide promising avenues to circumvent these difficulties. 

## 4. Antibody Therapy

Because of their high specificity, versatility, and relatively low cost, monoclonal antibodies have become the most utilized approach for immunomodulation. Immunoglobulin G (IgG) antibodies are designed to target specific antigens and are composed of both a constant and variable domain, which are present within the light and heavy chains of the immunoglobulin [39]. The use of antibodies to precipitate immunomodulatory action involves selective binding to components of the immune recognition pathway, where the monoclonal antibody coheres to the host immune cells or the tumor to facilitate immune cell recognition or block tumor cell immune evasion. In cancer therapies, the variable domain of monoclonal antibodies is modified to precisely enable targeting and binding to specific receptors on either the tumor cells or immune cells. This approach offers significant versatility; aside from immunomodulation, antibody-based therapy has also been used in directly cytotoxic ways. From antibody–chemotherapy conjugates that allow for tumor-specific delivery to blocking proliferative receptors on tumors such as anti-EGFR/VEGF, antibody therapy can enhance chemotherapeutic toxicity or prohibit active tumorigenesis [40,41]. In PDAC, however, a meta-analysis of four randomized controlled trials utilizing monoclonal antibody EGFR-inhibitor cetuximab showed no statistically significant improvements in clinical outcomes when compared to standard of care in patients with stage II–IV cancer treated with palliative intent [42]. As a result, there is much focus on antibody-based immunomodulation through immune checkpoint inhibition. Unfortunately, these antibody-based immune checkpoint inhibitors have also been historically challenged with low response rates in non-dMMR pancreatic cancer patients, as seen by the 0% response rate in PD-L1 positive PDAC [43]. To circumvent this, antibody therapies are often tested as combination regimens with chemotherapy as a mechanism of synergistic tumor toxicity via both the cytotoxic effects of chemotherapy alongside a conjoined enhanced immune recognition of tumor cells. 

In addition to the low response rate, antibody-based immunomodulation is associated with significant adverse effects in patients with both responsive and nonresponsive solid tumors. By blocking the immune checkpoint pathway, the immune system becomes more likely to also target normal cells, leading to a range of autoinflammatory side effects such as hepatitis, pneumonitis, and thyroid dysfunction being the most prominent [44]. To address these challenges, a novel approach currently being studied in prostate cancer and acute myeloid leukemia involves the use of a bispecific T-cell engager. This specific molecule possesses binding domains for both the T cell and tumor-specific antigens, thereby enhancing specificity by enhancing T-cell proximity and recognition specifically to tumor cells [45]. The clinical applicability of this approach has shown promising clinical response in small cell lung cancer (SCLC), where anti-CD3 x anti-delta-like ligand 3 (anti-DLL3) bispecific antibody tarlatamab was given FDA approval for platinum-treatment refractory SCLC [46]. These results showcase the considerable potential of bispecific antibodies in solid tumors. 

## 5. Vaccine Therapy

Cancer vaccination is a relatively novel approach, with the most prominent use involving prophylactic vaccination targeting high-risk strains of the human papillomavirus to prevent cervical cancer and the hepatitis B vaccine to prevent hepatocellular carcinoma [47]. In contrast, therapeutic vaccines are designed to activate the host immune system against existing malignant tumors. Therapeutic antitumor vaccines (TAVs) are currently under development for use in patients with ovarian (Gemogenovatucel-T/Vigil), prostate (Sipuleucel-T/Provenge), pancreatic, and several other solid tumor-associated cancers, including melanoma (Talimogene laherparepvec/Talimogeme laherparepvec) [48,49,50,51]. Specific mechanisms for the development and delivery of TAVs vary; however, each approach typically begins with obtaining a biopsy of the host tumor. The resulting TAVs, which are based on identifiable tumor-specific antigens, can be broadly classified as being cell-based (activated host immune cells or whole-cell/lysed tumor cells), peptide- or protein-based (recombinant or native tumor neoantigens), nucleotide-based (recombinant tumor DNA or mRNA), or microbe-based (recombinant viruses or bacteria) [52,53,54]. Additional TAVs may include adjuvants such as Interferon-α (IFN-α) or a granulocyte-macrophage colony-stimulating factor (GM-CSF), which are particularly important in enhancing the host’s response against tumor-derived antigens or recombinant neoantigens. This immunization strategy is intended to generate an adaptive immune response against both circulating and stationary tumor cells, theoretically leading to disease clearance. TAVs may be administered and supplemented with chemotherapy, radiotherapy, immune checkpoint inhibitors, or other immunotherapies, and may also be used in neoadjuvant or adjuvant settings before or after surgical intervention. 

Although TAVs have the potential to significantly improve outcomes in patients with advanced-stage cancer, several variables limit the effectiveness of TAVs and the patients who are considered viable candidates for vaccination. These include the presence of patient risk factors for adverse events, tumor heterogeneity from variable gene expression at a cellular level, and the degree of immunosuppression from the tumor microenvironment [55]. The tumor-specific factors that affect the viability of TAVs—namely, tumor heterogeneity and the tumor microenvironment—are particularly prevalent in pancreatic cancer, thus making the management of advanced disease cumbersome even without the use of TAVs. 

Preliminary data suggest that an advantage of using TAVs is their systemic efficacy as a personalized therapy, providing clinical benefit even in cases of advanced, metastatic, or otherwise treatment-resistant malignancies that are unresponsive to first-line therapy [52,55]. Currently, many ongoing trials are comparing the use of TAVs to respective standards of care for treating various solid tumors, particularly in patients with advanced disease. Although many of these studies are in the early stages, and data remains limited, what has been published has generally been promising. For example, adjuvant use of an autologous tumor cell-Bacillus Calmette–Guérin (BCG) vaccine (OncoVax)—irradiated tumor cells mixed with fresh-frozen Mycobacteria of the TICE strain of BCG—was found to be particularly effective when utilized in patients with moderate- to advanced-stage colorectal cancer [56]. Additionally, several other studies have exemplified the efficacy of Gemogenovatucel-T—a TAV that consists of autologous tumor cells transfected with a plasmid containing the GM-CSF gene, an immune-stimulatory cytokine, and a bifunctional short hairpin construct that inhibits furin—in treating ovarian cancer patients [57]. Last, although cell-based TAVs have arguably been the most common type utilized both during trials and in the clinical setting, mRNA-based TAVs have recently become a popular technique for use in patients with aggressive or treatment-resistant solid tumors, with promising results in treating melanoma [51].

In PDAC, recent advancements with mRNA vaccines have shown promising results in patients with resectable tumors, particularly when combined with anti-PD-L1 inhibition and adjuvant mFOLFIRINOX (Table 1; NCT04161755). Rojas et al. demonstrated in a small cohort of 16 patients that this combination induced high-magnitude, neoantigen-specific T-cell responses in 8 of the 16 patients. All 8 responders presented with no progression of disease at an 18-month follow-up, a significantly longer progression-free survival when compared to patients who did not respond to this combination [13]. The most significant limitation of this study was the lack of diversity in the patient population. Additionally, all patients included in the study were at a resectable stage of disease, already presenting with curative options in the form of surgery. In contrast, the low survival rates in pancreatic cancer are greatly attributed to metastatic disease at the time of diagnosis [58]. Therefore, further investigation into the ability of these neoantigen-recognizing T cells to target circulating cancer cells in the bloodstream, as well as their capability to identify and eliminate metastatic lesions, would be pivotal for extending the application of this promising approach to a broader population of pancreatic cancer patients. Overall, these findings suggest that TAVs hold significant potential as a viable therapeutic option in the ongoing fight against PDAC.

**Table 1:** Ongoing Immunotherapy-Based Trials in PDAC. All data acquired from ClinicalTrials.gov.

NCT	Phase	Intervention	Study Participant	Study Initiation
NCT00669734	I	Single Arm: Falimarev (recombiant fowlpox CEA-expressing viral vector vaccine) + Inalimarev (recombinant vaccinia CEA-expressing viral vector vaccine) + Sargramostim	*n* = 18 locally advanced or metastatic PDAC	2010–
NCT01595321	II	Arm A: SBRT + FOLFIRINOX Arm B: SBRT + modified FOLFIRINOX Arm C: CY + GVAX + SBRT + modified FOLFIRINOX	*n* = 19 surgically resected PDAC with no prior treatment	2012–
NCT02451982	II	Arm A: GVAX + CY Arm B: GVAX + Nivolumab + CY Arm C: GVAX + Nivolumab + Urelumab (anti-CD137) + CY Arm D: Nivolumab + BMS-986253 (anti-IL8)	*n* = 76 surgically resectable PDAC	2016–
NCT03104439	II	Single Arm: Nivolumab + Ipilimumab + Radiation	*n* = 80 CRC and PDAC with prior chemotherapy treatment	2017–
NCT03193190	I, II	Arm A: GnP Arm B: Atezolizumab (anti-PD-L1) + Selicrelumab (agonist CD40 Ab) + GnP Arm C: Atezolizumab + Bevacizumab (anti-VEGF-A) + GnP Arm D: Atezolizumab + AB928 (dual adenosine receptor antagonist) + GnP Arm E: Atezolizumab + Tiragolumab (anti-TIGIT) + GnP Arm F: Atezolizumab + Cobimetinib (Anti-MEK) Arm G: Atezolizumab + PEGPH20 Arm H: Atezolizumab + BL-8040 (CXCR4 antagonist) Arm I: Atezolizumab + RO6874281 (immunocytokine-targeting FAP) Arm J: Atezolizumab + Tocilizumab (anti-IL-6 mAb) + GnP	*n* = 340 metastatic PDAC with either no prior treatment (Arm 1) or disease progression following first-line systemic therapy (Arm 2)	2017–
NCT03080974	II	Single Arm: IRE + adjuvant Nivolumab	*n* = 10 locally advanced PDAC	2017–
NCT03323944	I	Single Arm: anti-mesothelin CAR vector-transduced autologous T-lymphocytes	*n* = 18 unresectable or metastatic PDAC	2017–
NCT03269526	I, II	Single Arm: anti-EGFR-armed activated T cells (EGFR BATs) + SoC	*n* = 22 locally advanced or metastatic pancreatic cancer who have received at least one dose of first-line chemotherapy	2017–
NCT03153410	I	Single Arm: CY + GVAX + Pembrolizumab + IMC-CS4 (anti-CSF1R Ab)	*n* = 12 borderline resectable PDAC	2018–
NCT03607890	II	Arm A: Nivolumab + Relatimab (Anti-LAG-3 Ab), coadministration Arm B: Nivolumab + Relatimab, sequential administration	*n* = 42 metastatic or locally advanced PDAC, received prior PD-1 therapy, mismatch repair-deficient disease	2018–
NCT03257761	I	Single Arm: Durvalumab + guadecitabine (antimetabolite)	*n* = 55 advanced HCC, PDAC, BC	2018–
NCT03563248	II	Arm A: FOLFIRINOX + SBRT + surgical resection Arm B: FOLFIRINOX + Losartan + SBRT + surgical resection Arm C: FOLFIRINOX + Losartan + Nivolumab + SBRT + surgical resection Arm D: FOLFIRINOX + Nivolumab + SBRT + surgical resection	*n* = 168 borderline resectable and locally advanced PDAC	2018–
NCT03404960	I, II	Arm A: Niraparib (PARP inhibitor) + Nivolumab Arm B: Niraparib + Ipilimumab	*n* = 104 advanced PDAC that has not progressed on platinum-based therapy	2018–
NCT03496662	I, II	Arm A: BMS-813160 (CCR2/CCR5 Inhibitor) + Nivolumab + GnP Arm B: GnP	*n* = 40 borderline resectable and locally advanced PDAC	2018–
NCT03592888	I	Single Arm: Mature dendritic cell (mDC3/8) vaccine primer and booster	*n* = 29 resected PDAC with KRAS(G12D), KRAS(G12V), KRAS(G12R), KRAS(G12C-mutated), HLA-A02, HLA-A03, HLA-A11, HLA-B07, HLA-C08	2018–
NCT03006302	II	Arm A: Epacadostat (IDO1 inhbitor) + Pembrolizumab + CRS-207 + CY + GVAX, on different dosages Arm B: Epacadostat + Pembrolizumab + CRS-207 on different dosages	*n* = 41 metastatic PDAC that has progressed on prior chemotherapy	2018–
NCT03829501	I, II	Arm A: KY1044 (anti-ICOS) Arm B: KY1044 +Atezolizumab	*n* = 280 metastatic solid tumors, including PDAC	2019–
NCT03970252	I	Single Arm: Nivolumab + mFOLFIRNOX pre-surgery	*n* = 28 borderline resectable PDAC	2019–
NCT03682289	II	Arm A: Ceralasertib (ATR kinase inhibitor) Arm B: Ceralasertib + Olaparib (PARP inhibitor) Arm C: Ceralasertib + Durvalumab	*n* = 89 locally advanced or metastatic solid tumors, including PDAC	2019–
NCT03816358	I	Arm A: Anetumab Ravtansine (anti-mesothelin) + Nivolumab Arm B: Anetumab Ravtansine + Ipilimumab + Nivolumab Arm C: Anetumab Ravtansine + Nivolumab + Gemcitabine Hydrochloride	*n* = 74 recurrent, unresectable, or metastatic mesothelin-positive PDAC	2019–
NCT04137536	I	Single Arm: EGFR BATs	*n* = 7 metastatic PDAC already treated with first-line standard chemotherapy	2019–
NCT03745326	I, II	Single Arm: anti-KRAS G12D murine TCR PBL cells + CY + Fludarabine (antimetabolite) + Aldesleukin (recombinant IL-2)	*n* = 70 metastatic or unresectable cancers with G12D mutated KRAS, NRAS or HRAS, HLA-A*11:01 positive, no prior therapy or nonresponders; PDAC, GI cancer, gastric cancer, colon cancer, rectal cancer	2019–
NCT03806309	II	Arm A: OSE2101 (T cell epitope-based vaccine) + FOLFIRI Arm B: FOLFIRI	*n* = 106 HLA-A2 patients with locally advanced or metastatic PDAC not amenable to surgery	2019–
NCT04161755	I	Single Arm: RO7198457 (Personalized Tumor Vaccine) + Atezolizumab + mFOLFIRNOX	*n* = 29 resectable or radiographically resectable PDAC	2019–
NCT03767582	I, II	Arm A: GVAX + Nivolumab + SBRT + CCR2/CCR5 dual antagonist Arm B: Nivolumab + SBRT + CCR2/CCR5 dual antagonist	*n* = 30 locally advanced unresectable PDAC	2019–
NCT04390763	II	Arm A: NIS793 (anti-TGF-β) + Spartalizumab + GnP Arm B: NIS973 + GnP Arm C: GnP	*n* = 164 treatment naive, metastatic PDAC	2020–
NCT04477343	I	Single Arm: SX-682 (CXCR1/2 inhibitor) + Nivolumab as maintenance	*n* = 20 metastatic PDAC with 16+ weeks first-line chemo without evidence of progression	2020–
NCT04612530	I	Arm A: Nivolumab Arm B: Nivolumab + IRE Arm C: Nivolumab + IRE + TLR ligand (CpG)	*n* = 18 primary oligometastatic PDAC	2020–
NCT04493060	II	Single Arm: Niraparib (PARP inhibitor) + Dostarlimab (anti-PD-1)	*n* = 22 germline or somatic BRCA and PALB2 metastatic PDAC	2020–
NCT04672434	I	Arm A: Sym024 (anti-CD73), tested at different dosages Arm B: Sym024 + Sym021 (anti-PD-L1), tested at different dosages	*n* = 48 locally advanced or metastatic solid tumors, including PDAC	2020–
NCT04365049	observational	Arm A: Camrelizumab (PD-1) + Radiotherapy + GnP Arm B: GnP	*n* = 100 locally advanced PDAC	2020–
NCT04666740	II	Arm A/B: Pembrolizumab + Olaparib in patients with homologous recombination mutations, with stable or responding disease on platinum therapy Arm C: Pembrolizumab + Olaparib in patients without homologous recombination mutations with platinum-sensitive disease	*n* = 63 metastatic PDAC with responding disease on platinum-based treatment or homologous recombination gene deficiency	2020–
NCT04581473	I, II	Single Arm: Claudin 18.2-targeting autologous CAR-T cell injection (CT041)	*n* = 192 advanced PDAC or GEA positive for Claudin 18.2 who have failed at least 2 prior lines treatment, or patients with pathologically diagnosed advanced PDAC who have failed at least 1 prior line treatment	2020–
NCT04157127	I	Single Arm: Th-1 DC immunotherapy (autologous DC) vaccine	*n* = 43 potentially resectable PDAC following completion of standard chemotherapy	2020–
NCT04627246	I	Single Arm: PEP-DC (autologous DC vax loaded with personal peptides) + Nivolumab + SoC chemotherapy	*n* = 12 resectable PDAC	2020–
NCT04753879	II	Single Arm: Low-dose chemotherapy GAX-CI followed by Olaparib + Pembrolizumab	*n* = 38 untreated metastatic PDAC	2021–
NCT04548752	II	Arm A: Olaparib Arm B: Olaparib + Pembrolizumab	*n* = 88 metastatic PDAC with germline BRCA 1/2 mutation	2021–
NCT04940286	II	Single Arm: Durvalumab + Oleclumab + GnP	*n* = 30 resectable or borderline resectable PDAC	2021–
NCT04802876	II	Arm A: Spartalizumab in patients with high PD-1 expression Arm B: Spartalizumab in patients with low PD-1 expression Arm C: Tislelizumab (anti-PD-1) in patient with high PD-1 expression	*n* = 184 PD1-high mRNA expressing solid tumors, including PDAC	2021–
NCT04888312	Ib, II	Single Arm: Mitazalimab (anti-CD40) + FOLFIRINOX	*n* = 94 metastatic PDAC	2021–
NCT04887805	II	Single Arm: Pembrolizumab + Lenvatinib (TKI inhibitor)	*n* = 28 advanced unresectable PDAC	2021–
NCT05000294	I, II	Single Arm: Atezolizumab + Tivozanib (VEGF inhibitor) in immunologically cold tumors	*n* = 29 metastatic immunologically cold tumors, including PDAC	2021–
NCT04146298	I, II	Single Arm: Mutant KRAS G12V-specific TCR transduced T-cell therapy	*n* = 30 locally advanced or metastatic PDAC with KRAS G12V mutation and HLA-A*11:01	2021–
NCT05239182	II	Single Arm: 9-ING-41 (GSK-3β inhibitor) + Retinfanlimab (anti-PD-1) + GnP	*n* = 32 previously untreated metastatic PDAC	2022–
NCT05052723	II	Single Arm: Pembrolizumab + Cabozantinib	*n* = 21 metastatic PDAC progressed on SoC	2022–
NCT05132504	II	Single Arm: mFOLFIRINOX + Pembrolizumab followed by surgery	*n* = 30 resectable PDAC	2022–
NCT05088889	I	Single Arm: Ipilimumab + Nivolumab + SBRT + low dose irradiation	*n* = 10 metastatic PDAC	2022–
NCT05102721	I, II	Single Arm: Avelumab (anti-PD-1) + Pepinemab (anti-*SEMA4D*)	*n* = 48 metastatic PDAC after progression on first-line chemotherapy	2022–
NCT05239143	I	Single Arm: P-MUC1-Allogenic CAR-T cells (targeting the Mucin 1 antigen)	*n* = 180 advanced or metastatic epithelial-derived solid tumors refractory to SoC, including PDAC	2022–
NCT05194735	I, II	Arm A: TCR-T cell (sleeping beauty transposon/transposase to express TCRs against neoantigens) Arm B: TCR-T cell + IL 2	*n* = 180 with solid tumors who are TCR-applicable, completed HLA typing, and progressed on at least SoC therapy, including PDAC	2022–
NCT05014776	II	Single Arm: Tadalafil (PDE5 inhibitor) + Pembrolizumab + Ipilimumab + CRS-207 (Listeria vaccine)	*n* = 17 previously treated metastatic PDAC	2022–
NCT06005493	I, II	Arm A: AZD5863 (CLDN18.2) Intravenous Arm B: AZD5863 Subcutaneous	*n* = 200 locally advanced or metastatic tumor expressing Claudin 18.2; PDAC, gastric cancer, GEA	2023–
NCT05482893	I, II	Arm A: PT886 (CLDN18.2, CD47) dose escalation Arm B: PT886 (ClLDN18.2, CD47) dose expansion Arm C: PT886 + GnP Arm D: PT886 + Pembrolizumab + oxaliplatin + leucovorin + Fluorouracil + capecitabine	*n* = 114 unresectable or metastatic PDAC and GEA	2023–
NCT05604560	II	Single Arm: Tislelizumab and SX-682 (CXCR1/2 inhibitor)	*n* = 25 patients with resectable PDAC	2023–
NCT05945823	II	Single Arm (For PDAC): Pembrolizumab + Futibatinib (FGER1-4 inhibitor) + mFOLFIRINOX	*n* = 66 locally advanced or metastatic solid tumors, including PDAC	2023–
NCT05630183	II	Arm A: Botensilimab (CTLA-4 inhibitor) + GnP Arm B: GnP	*n* = 78 metastatic PDAC with progression on FOLFIRINOX	2023–
NCT06060405	II	Single Arm: Durvalumab and Oleclumab	*n* = 22 resectable PDAC	2023–
NCT06051851	II	Arm A: Penpulimab (anti-PD-1) and Anlotinib (multitargetting TKI) + GnP Arm B: GnP	*n* = 177 untreated metastatic PDAC	2023–
NCT05558982	II	Single Arm: BXCL701 (DPP inhibitor) + Pembrolizmab	*n* = 43 metastatic PDAC refractory to SoC	2023–
NCT05846516	I	Arm A: VSV-GP154 (chimeric oncolytic vesicular stomatitis virus vaccine with undisclosed peptides) + ATP150 (undisclosed protein vaccine) + ATP152 (undisclosed protein vaccine) Arm B: VSV-GP154 + ATP150 + ATP152 + Ezabenlimab (anti-PD-1)	*n* = 85 KRAS G12D or KRAS G12V-mutated advanced or metastatic PDAC	2023–
NCT05968326	II	Arm A: Autogene Cevumeran (individualized neoantigen vaccine) + Atezolizumab + mFOLIRINOX Arm B: mFOLFIRINOX	*n* = 260 resected T1–T3, N0–N2, M0 PDAC with no prior systemic treatment	2023–
NCT05927142	I, II	Single Arm: Durvalumab with Rintatolimod (TLR-3 agonist)	*n* = 43 stable metastatic PDAC	2024–
NCT06158139	I	Single Arm: Autologous CAR-T targeting the B7-H3 antigen	*n* = 27 B7-H3 antigen-positive PDAC refractory to SoC	2024–
NCT06015724	II	Single Arm: Daratumumab (anti-CD38) + KRAS vaccine + Nivolumab	*n* = 54, advanced PDAC or NSCLC with mutated KRAS G12A, C, D, R, S, V, or KRAS G13D and failed one prior treatment	2024–

Ab: antibody, ATR: ataxia telangiectasia and Rad30-related, B7-H3: B7 homolog 3 protein, BATs: bispecific antibody-armed activated T cells, BC: breast cancer, BRCA: breast cancer gene, CEA: carcinoembryonic antigen, CRC: colorectal cancer, CSF1R: colony-stimulating factor 1 receptor, DPP: dipeptidyl peptidases, EGFR: epidermal growth factor receptor, FAP: fibroblast activation protein–a, FGFR: fibroblast growth factor receptor, FOLFIRI: 5-fluorouracil, leucovorin, irinotecan, GAX-CI: gemcitabine, nab-paclitaxel, capecitabine, cisplatin, irinotecan, GEA: gastroesophageal adenocarcinoma, GI: gastrointestinal, GSK-3β: glycogen synthase kinase-3β, HCC: hepatocellular carcinoma, HLA: human leukocyte antigen, HRAS: Harvey rat sarcoma virus, ICOS: inducible T-cell co-stimulator, IDO1: indoleamine 2,3-dioxygenase-1 inhibitor, IL: interleukin, LAG-3: lymphocyte activation gene-3, mAb: monoclonal antibody, MEK: mitogen-activated extracellular signal-regulated kinase, mFOLFIRINOX: modified FOLFIRINOX, MUC1: Mucin 1, NRAS: neuroblastoma rat sarcoma virus, PARP: poly(ADP-ribose) polymerase, PALB2: partner and localizer of BRCA2, PBL: peripheral blood lymphocyte, SEMA4D: semaphorin 4D, TCR: T-cell receptor, TIL: tumor-infiltrating lymphocytes, TKI: tyrosine kinase inhibitor, TGF-β: transforming growth factor β, TIGIT: T-cell immunoglobulin and ITIM domain, VEGF: vascular endothelial growth factor.

## 6. Cellular Therapy

Cellular therapy represents a broad pharmacological category involving the autologous or allogeneic transfer of cellular material into a patient to enhance the immune system’s ability to efficiently repair tissue or combat cancers, autoimmune diseases, or infectious diseases [59,60]. Adoptive cell therapy used in cancer treatment involves the transfer of immune cells for direct therapeutic benefit. The method has employed various actors, including DCs, NK cells, cytokine-induced killer cells, lymphokine-activated killer cells, and macrophage killer cells [59,61]. Genetic recombination of this approach allows for precise targeting of tumor-specific responses, enabling high specificity with low toxicity, decreased opportunity for tumor escape or therapeutic resistance, and the ability to proliferate and navigate through tumor microenvironments [59,62]. FDA-approved cellular therapies include agents that specifically target hematogenous malignancies and advanced melanoma as of February 2024, extensive-stage SCLC as of June 2024, and extensive synovial sarcoma as of August 2024, whereas ongoing research includes the focus of expanding applicability to solid tumors [63]. However, significant challenges remain, including the barriers to cellular therapy in solid neoplasms, including the tumor microenvironment, the heterogeneity of tumor antigens, and the requirement for intimate structural knowledge of antigen-presenting cells for effective target design [62,64,65]. Despite these challenges, cellular therapy has a high potential to revolutionize the approaches for solid cancer treatment. 

Chimeric antigen receptor T-cell (CAR-T) therapy involves the autologous harvest of T lymphocytes and subsequent genetic modification to accurately identify and eliminate malignant cells expressing the chosen target antigen [59,65]. CAR-T cells have proven to be advantageous in providing both an immediate and persistent effect because they continue to proliferate via clonal expansion [66]. There are four fundamental components of a CAR-T cell, with an extracellular targeting domain composed of a variable antibody fragment allowing for specific tumor antigen recognition and MHC-independent binding. The other three components include a hinge region providing flexibility of the antigen-binding domain, a transmembrane domain enabling increased stability and anchoring to the target cell, and at least one intracellular signaling domain to enhance activation and lymphocytic proliferation [65,67,68]. Since their inception, CAR-T cells have advanced through five generations to improve proliferation and accuracy while decreasing toxicity.

Currently, second-generation CAR-T cells are approved for use in B-cell malignancies, with the addition of a second costimulatory molecule allowing for improved T-cell response and persistence compared to the initial generation. An additional costimulatory domain added in the third generation enables more effective and accelerated tumor clearance. The fourth generation, termed armored CAR-T cells or TRUCKs (T-cells Redirected for antigen-Unrestricted Cytokine-initiated Killing), includes an encoded interleukin inducer to overcome the barrier of the immunosuppressive tumor microenvironment, suggesting broader applicability in solid tumors [65,67,68,69]. Fifth-generation CAR-T cells are in the early stages of development, including the addition of a signal transducer and activator of transcription 3 (STAT3) binding site to a monovalent CAR-T cell to engage the Janus kinase (JAK)-STAT pathway, promoting thorough stimulation of the immunological response [70]. The efforts to enhance CAR-T cell performance in PDAC and other solid tumors are multifaceted and involve increasing efficacy by addressing the challenges posed by stromal barriers, immunosuppressive TME, and antigen escape. 

Commonly expressed stromal elements, such as mesothelin, have been used as the extracellular targeting domain to improve stromal penetration [67]. Studies investigating non-systemic delivery methods in glioblastoma aim to deliver CAR-T cells more directly to malignancy regions while reducing systemic toxicity [65]. Furthermore, using armored CAR-T cells with cytokine activity has been shown to combat the immunosuppressive TME by recruiting local immune cells, and their synergistic application with other immunotherapies could theoretically increase their effectiveness [65,71]. Additionally, research into the development of “tandem CAR-T cells”, which includes two distinct antibody fragments, has been proven to enhance the recognition and destruction of tumor cells with heterogeneous antigen expression across various solid cancers, including gastrointestinal malignancies such as colon, gastroesophageal adenocarcinoma (GEA), and liver cancers [65,72]. These advancements highlight the ongoing process of optimizing CAR-T cell therapies to overcome the challenge of treating solid tumors. 

## 7. Discussion

Though the small subset of dMMR PDAC tumors respond well to PD-L1 inhibitors, immunomodulatory antibodies have not shown the same caliber of widespread clinical benefit in PDAC when compared to other solid tumors such as non-small cell lung cancer (NSCLC) [7,73]. In looking at broader trends in clinical trial results utilizing antibody-based immune checkpoint inhibitors alone, of the 15 completed antibody-based immunomodulatory trials included in this review, only one showed clinically significant improvements from the control (Table 2). The other 14 trials were either halted prematurely because of poor response, completed with worse patient outcomes as compared to standard of care, or completed with no significant differences in patient outcomes when compared to standard of care. Focusing on anti-PD-1/anti-PD-L1 therapies, both monotherapies and combined immunomodulatory antibody-based approaches demonstrated remarkedly low response rates across nine trials listed in Table 2 (NCT02558894, NCT02503774, NCT02646748, NCT03250273, NCT03723915, NCT03549000, NCT03634332, NCT04060342, and NCT05061017) [74,75,76,77,78,79,80,81,82]. However, when used as a combination therapy with chemotherapeutic agents, anti-PD-1 therapy has shown some promise [83]. For example, a trial utilizing combination therapy with nivolumab, a PD-1 inhibitor, and GnP demonstrated a clinically significant improvement in overall survival without a difference in toxicity compared to the standard of care (Table 2; NCT03214250) [83]. It is not yet clear mechanistically what led to the observed synergistic effect when alternative antibodies using a similar mechanism of action did not demonstrate clinical benefit. Therefore, further investigation regarding the role of nivolumab and the PD-1 checkpoint when administered in combination with GnP is necessary, and a nivolumab + gemcitabine intervention group is being tested in another trial to further assess this synergism (Table 1; NCT03816358). Currently, several ongoing antibody-based trials are assessing the efficacy of nivolumab-paired other chemotherapeutics, most notably the other primary first-line regimen FOLFIRINOX (Table 1; NCT03970252, NCT04612530, NCT03563248, NCT03806309, and NCT05088889). 

**Table 2:** Completed Antibody-Based Immunomodulatory Trials in PDAC. * = data acquired from American Society of Clinical Oncology (ASCO) Publications, ^+^ = data acquired from PubMed or peer-reviewed publication, ^#^ = data acquired from ClinicalTrials.gov. The confidence interval of 95% is included in parenthesis when available.

NCT	Phase	Intervention	Study Participant	Study Duration	Clinical Outcomes
NCT01473940 ^#^, [84]	Ib	Gemcitabine Hydrochloride + Ipilimumab (anti-CTLA-4) 3 mg/kg (Arm A) or 6 mg/kg (Arm B)	*n* = 20, unresectable PDAC	2012–2018	mPFS: 2.52 mo in Arm A (0.789–4.83), 3.86 mo in Arm B (0.756–22.42), OS: 5.72 mo in Arm A (1.61–22.81), 8.99 mo in Arm B (0.75–30.05)
NCT02558894 ^#^, [74]	II	Arm A: Durvalumab (anti-PD-L1) and tremelimumab (anti-CTLA-4) Arm B: Durvalumab	*n* = 65, metastatic PDAC	2015–2017	ORR: 3.1% in Arm A (0.08–16.22), 0% in Arm B (0–10.58) PFS: 1.5 mo in Arm A (1.2–1.5), 1.4 mo in Arm B (1.3–1.5) mOS: 3.1 mo in Arm A (2.2–6.1), 3.6 mo in Arm B (2.7–6.1)
NCT02503774 ^+^, [75]	I	Single Arm: Oleclumab (anti-CD73) + durvalumab	*n* = 192 advanced solid tumor, *n* = 42 advanced PDAC	2015–2021	PFS-6 mo (PDAC): 13.2% ORR (PDAC): 4.8%
NCT02305186 *, [85]	I, II	Arm A: Pembrolizumab (anti-PD-L1) + neoadjuvant CRT Arm B: Neoadjuvant CRT	*n* = 37, resectable/borderline resectable PDAC	2015–2022	OS: 27.8 mo A vs. 24.3 mo B (*p* = 0.68) mRFS: 18.2 mo A vs. 14.1 mo B (*p* = 0.41)
NCT02527434 *, [86]	II	Single Arm: Tremelimumab	*n* = 20 metastatic PDAC with prior first-line chemotherapy	2015–2023	ORR: 0% (0–16.8) mOS: 3.98 mo (2.83–5.42)
NCT02583477 ^#^, [87]	I, II	Durvalumab + AZD5069 (CXCR2 inhibitor)	*n* = 20 metastatic PDAC	2016–2018	ORR: 5.6% (0.58–19.95) DCR-12: 5.6 mo mPFS: 1.6 mo (1.29–1.69)
NCT02646748 ^+^, [76]	I	Single Arm: Pembrolizumab + itacitinib (JAK inhibitor)	*n* = 159 advanced solid tumors, *n* = 8 advanced PDAC	2016–2020	ORR (PDAC): 12.5%
NCT03250273 ^#^, [77]	II	Single Arm for PDAC: Nivolumab (anti-PD-1) + entinostat (HDAC inhibitor)	*n* = 30 metastatic PDAC	2017–2020	ORR: 11.1% OS: 2.729 mo (1.84 to 5.64) PFS6: 0.067 (0.017–0.254)
NCT03214250 ^#^, [88]	I, II	Arm A: GnP + nivolumab Arm B: GnP + sotigalimab (CD40 agonist) Arm C: GnP + sotigalimab + nivolumab	*n* = 105 previously untreated metastatic PDAC	2017–2022	One-year OS: 0.577 in Arm A (0.384–0.729), 0.481 in Arm B (0.309–0.634), 0.413 in Arm C (0.244–0.575) PFS: 6.37 mo A (5.19–8.80), 7.26 mo B (5.36–9.23), 6.74 mo C (4.17–9.79) ORR: 50% A (32.43–67.57), 33% B (18.56–50.97), 31.4% C (16.85–49.29)
NCT03723915 ^#^, [78]	II	Single Arm: Pembrolizumab and pelareorep (oncolytic reovirus)	*n* = 17, advanced PDAC	2018–2019	ORR: 8.33% mPFS: 1.87 mo (1.61–7.20) mOS: 6.21 mo (2.63–26.08) Terminated due to interim analysis criteria not met
NCT03549000 ^#,^*, [79]	I	Arm A: NZV930 (anti-CD73) Arm B: spartalizumab (anti-PD-L1), NIR178 (A2A receptor inhibitor)	*n* = 127 advanced solid tumors, *n* = 11 advanced PDAC	2018–2022	Terminated early because of poor interim analysis of treatment efficacy
NCT03611556 ^#,^* [89]	I, II	Arm A: GnP Arm B: Oleclumab + GnP Arm C: Oleclumab + durvalumab + GnP	*n* = 195 metastatic PDAC	2018–2022	PFS: 5.6 mo B (3.5–7.5), 7.5 mo C (5.5–10.9) DoR: 12.9 mo B(2.2–NA), 9.5 mo C (5.7–12.0) OS: 8.9 mo B (6.9–11.5), 12.9 mo C (10.1–15.3)
NCT03634332 *, [80]	II	Single Arm: PEGPH20 and pembrolizumab	*n* = 8, metastatic PDAC + high hyaluronic acid	2019–2021	mOS: 7.2 mo (1.2–11.8) mPFS: 1.5 mo (0.9–4.4) Halted accrual early due to lack of response
NCT04060342 ^#^, [81]	I	Arm A: GB1275 (CD11b modulator) Arm B: GB1275 + pembrolizumab Arm C: GB1275 + GnP	*n* = 61, metastatic PDAC	2019–2022	No clear benefit of GB1275 was observed either as monotherapy or in combination with pembrolizumab
NCT05061017 ^+^, [82]	II	Arm A: Nivolumab + pixatimod (TLR-9 activator) Arm B: Nivolumab + pixatimod + CY	*n* = 58 solid tumor patients, *n* = 18 PDAC	2021–2024	ORR (PDAC): 0%

anti-X: antibody-targeting X, CRT: chemoradiotherapy, CTLA-4: cytotoxic T-lymphocyte associated protein 4, CXCR2: C-X-C motif chemokine receptor 2, CY: Cyclophosphamide, DCR: disease control rate, DoR: duration of response, GnP: gemcitabine and nab-paclitaxel, HDAC: histone deacetylase, JAK: Janus kinase, mo: month, mOS: median overall survival, mPFS: median progression-free survival, mRFS: median recurrence-free survival, NA: not available, NCT: national clinical trial, ORR: objective response rate, OS: overall survival, PDAC: pancreatic ductal adenocarcinoma, PD-1: programmed cell death protein 1, PDL-1: programmed death ligand 1, PEGPH20: pegylated recombinant human hyaluronidase, PFS: progression-free survival, PFS6: progression-free survival at 6 months, TLR: toll-like receptor.

Anti-CD73-based approaches have faced similar challenges to anti-PD-1/anti-PD-L1 therapies. Of the three clinical trials utilizing anti-CD73, all demonstrated either poor response rates or no significant improvements in OS and ORR in PDAC patients compared to SoC (Table 2: NCT03611556, NCT02503774, and NCT03549000) [75,79,89]. In trial NCT02503774, CD73-positive tumor cells were stained before treatment, and among the two patients who responded to anti-C73 therapy, both had over 80% of their tumor cells staining positive for CD73 [75]. The trial NCT03611556 [89] tested the combination of oleclumab, an anti-CD73 monoclonal antibody, with durvalumab and chemotherapy in metastatic PDAC. Results from this study showed that the response rate in PDAC patients remained low, with no significant impact on OS or ORR observed. However, results from exploratory analyses indicated that patients with high CD73 expression levels showed some improvement in PFS and OS when treated with this combination therapy. These trials suggest that, similar to how dMMR tumors are more prone to anti-PD-1/anti-PD-L1 therapy, high CD73-expressing PDAC tumors could potentially serve as a biomarker for response to anti-CD73 therapy. 

**Figure 1:**
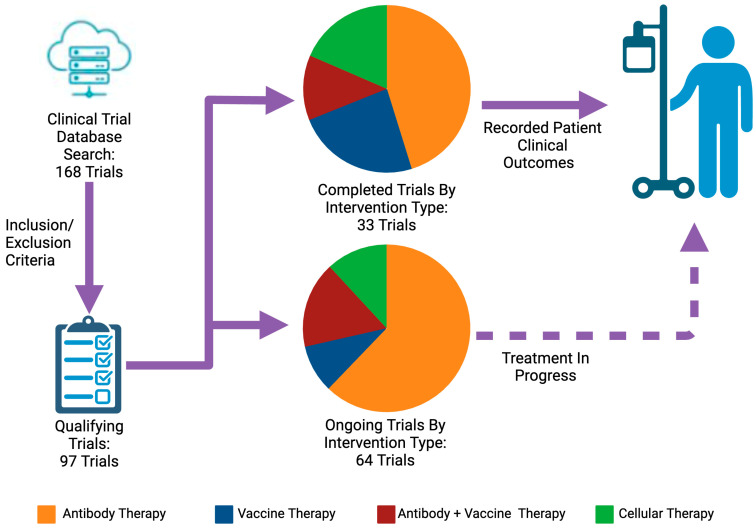
Current trends in immunotherapy in pancreatic cancer. The solid line indicates the trials with a completed status on ClinicalTrials.gov, and the dashed line represents the trials with an active status on ClinicalTrials.gov as of August 2024.

Although there is still potential for current monoclonal antibody-based therapies to improve patient survival that requires further investigation, most of these trials have failed to show significant improvement in clinical outcomes. Further research is required to possibly modify the fundamental approach regarding antibody-based therapies to transition to alternative targets in immune checkpoint recognition, such as pairing these inhibitors to vaccine-based therapies (Table 3). In tandem, looking to bispecific antibody-based approaches to further expand the ability to selectively block immune checkpoint recognition from tumoral cells to colocalize immune cells to tumor cells can mitigate the frequent inflammatory responses seen from these antibody-based immune checkpoint inhibitors and potentially improve response rates. Antibody-based immunomodulation continues to be the most trialed approach, looking at both completed and ongoing clinical trials included in this review (Figure 1). However, further expanding on cellular and vaccine therapies may yield promising results. Cellular and vaccine treatment provides an alternative approach to antibodies that may be potentially less hindered by the same resistance pathways.

**Table 3:** Completed Vaccine + Antibody-Based Trials in PDAC. * = data acquired from ASCO Publications; ^#^ = data acquired from ClinicalTrials.gov. The confidence interval of 95% is included in parentheses when available.

NCT	Phase	Intervention	Study Participant	Study Duration	Clinical Outcomes
NCT00836407 ^#^, [90]	I	Arm A: Ipilimumab alone Arm B: Ipilimumab + pancreatic cancer vaccine (allogenic pancreatic tumor cells transfected with a *GM-CSF* gene)	*n* = 30 advanced PDAC	2009–2012	OS: 3.6 mo Arm A (2.5–9.2) vs. 5.7 mo Arm B (4.3–14.7)
NCT01896869 ^#^, [91]	II	Arm A: Ipilimumab + pancreatic cancer vaccine Arm B: FOLFIRNOX	*n* = 82 metastatic PDAC treated with FOLFIRNOX with ongoing response or stable disease after 8–12 doses	2013–2019	OS: 9.38 mo Arm A (5.0–12.2) vs. 14.7 mo Arm B (11.6–20.0) PFS: 2.4 mo Arm A (1.87–2.53) vs. 5.55 mo Arm B (3.32–8.51) ORR: 2.9% Arm A vs. 10.3% Arm B
NCT02243371 ^#^, [92]	II	Arm A: CY + GVAX + CRS-207 + Nivolumab Arm B: CY + GVAX + CRS-207	*n* = 93 previously treated metastatic PDAC	2015–2017	OS: 5.88 mo Arm A (4.73–8.64) vs. 6.11 mo Arm B (3.52–7.00) PFS: 2.23 mo Arm A (2.14–2.33) vs. 2.17 mo Arm B (2.00–2.30)
NCT03161379 *, [93]	II	Single Arm: CY + Nivolumab + GVAX + SBRT	*n* = 31 borderline resectable PDAC	2018–2024	MPRR (<10% residual viable tumor): 35% mOS: 20.4 mo (18.2–NA)

CR: complete response, FOLFIRINOX: 5-fluorouracil, leucovorin, irinotecan, and oxaliplatin, MPRR: major pathologic response rate, SBRT: stereotactic body radiation therapy, yr: year.

Since 2002, there have been 31 studies investigating the safety and efficacy of TAVs in pancreatic cancer patients, with 14 of these studies currently ongoing. By far, the most utilized TAV regimens currently under investigation for pancreatic cancer patients are centered around the granulocyte-macrophage colony-stimulating factor (GM-CSF) gene-transfected tumor cell vaccine (GVAX)—utilized in 16 studies listed in Table 3 and Table 4 (NCT00389610, NCT03190265, NCT02004262, NCT01417000, NCT00084383, NCT02243371, NCT01896869, NCT00836407, and NCT03161379) [90,91,92,94,95,96,97,98], as well as in Table 1 (NCT01595321, NCT02451982, NCT03006302, and NCT03767582). This vaccine utilizes two irradiated allogeneic pancreatic tumor cell lines transfected with the *GM-CSF* gene to induce an adaptive immune response like attenuated viral/bacterial vaccines while simultaneously stimulating the immune system, ultimately resulting in host-derived targeting of the tumor. Other TAVs frequently used in pancreatic cancer clinical trials include CRS-207 (live-attenuated Listeria monocytogenes expressing human mesothelin) and recombinant peptide vaccines that are listed in Table 4 (NCT03190265, NCT02004262, and NCT01417000) [96,97,98]. Despite the theoretical potency of TAVs, the results of studies investigating their use against pancreatic cancer have been mixed. Although most studies have not published their findings, 5 of the 31 studies investigating TAVs in patients with pancreatic cancer were terminated because of lack of survival benefit or were completed and showed either no improvement in survival or decreased survival compared to the standard of care listed in Table 3 and Table 4 (NCT03190265, NCT00358566, NCT00425360, NCT02004262, and NCT01896869) [91,97,98,99,100]. Trials investigating the use of the telomerase reverse transcriptase vaccine, GV1001, in patients with pancreatic cancer resulted in negative findings disproportionately compared to other TAVs [101]. This is likely because of the particularly immunosuppressive tumor microenvironment that is characteristic of pancreatic cancer, as mentioned previously [11]. Even with a sensitized immune system, the inability of immune cells to infiltrate or function within the tumor microenvironment ultimately minimizes the effectiveness of TAVs in these patients. Thus, future consideration of patient-specific factors may be necessary to direct future TAV development and treatment parameters. 

**Table 4:** Completed Vaccine-Based Trials in PDAC. * = data acquired from ASCO Publications; ^+^ = data acquired from PubMed or peer-reviewed publications; ^#^ = data acquired from ClinicalTrials.gov. The confidence interval of 95% is included in parentheses when available. HR: hazard ratio, SoC: standard of care.

NCT	Phase	Intervention	Study Participant	Study Duration	Clinical Outcomes
NCT00084383 ^#^, [94]	II	Single Arm: GVAX following SoC	*n* = 60, surgically resected PDAC	2002–2006	OS: 24.8 (21.2–31.6) DFS: 17.3 mo (14.6–22.8)
NCT00358566 ^#^, [99]	III	Arm A: Gemcitabine Arm B: GV1001 (telomerase peptide vaccine) + gemcitabine	*n* = 360, locally advanced or metastatic PDAC	2006–2008	Terminated, preliminary data showed no survival benefit in the GV1001 group compared to the gemcitabine group.
NCT00425360 *, [100]	III	Arm A: Sargramostim (recombinant human GM-CSF) + GV1001 + capecitabine + gemcitabine hydrochloride concurrently Arm B: Capecitabine + gemcitabine hydrochloride with sequential GV1001 Arm C: Capecitabine + gemcitabine hydrochloride alone	*n* = 1062 locally advanced or metastatic PDAC	2006–2013	OS: 8.4 mo (7.3–9.7) in Arm A vs. 6.9 mo (6.4–7.6) in Arm B vs. 7.9 mo (7.1–8.8) in Arm C
NCT00389610 ^#^, [95]	II	Arm A: Previously vaccinated with GVAX, booster every 6 mo Arm B: GVAX naive, priming once a mo for 3 mo, every 6 mo afterwards	*n* = 56 surgically resected PDAC	2006–2022	OS: 80.5 mo in Arm A (22.5 to 187.8), 30.7 mo in Arm B (19.3 to 40.7) DFS after 16 years: 109.5 mo in Arm A (5.59 to NA), 13.7 in Arm B (5.55 to 25.1)
NCT01417000 ^#^, [96]	II	Arm A: CY + GVAX + CRS-207 (mesothelin-expressing LADD) Arm B: CY + GVAX	*n* = 93 previously treated metastatic PDAC	2011–2017	OS: 6.26 mo in Arm A (4.47–9.40) vs. 4.07 mo in Arm B (3.32–5.42)
NCT02261714 ^#^, [102]	I, II	Single Arm: TG01 (KRAS vaccine)/GM-CSF + gemcitabine	*n* = 32 surgically resected PDAC	2012–2019	OS: 33.3 mo (24.0–40.0) DFS: 16.1 mo (11.1–19.6)
NCT02004262 ^+^, [97]	II	Arm A: CY + GVAX + CRS-207 Arm B: CRS-207 Arm C: SoC	*n* = 213 previously treated metastatic PDAC	2014–2016	mOS: 3.7 mo in Arm A (2.9–5.3) vs. 5.4 mo in Arm B (4.2–6.4) vs. 4.6 mo in Arm C (4.2–5.7) HR: 1.17 (0.84–1.64)
NCT03190265 ^#^, [98]	II	Arm A: CY + Nivolumab + Ipilimumab + GVAX + CRS-207 Arm B: Nivolumab + Ipilimumab + CRS-207	*n* = 61 previously treated metastatic PDAC	2017–2023	ORR 0.0% in Arm A, 7.4% in Arm B

DFS: disease-free survival, GM-CSF: granulocyte macrophage colony-stimulating factor, GVAX: granulocyte-macrophage colony-stimulating factor gene-transduced tumor cell vaccine, HR: hazard ratio, KRAS: Kristen rat sarcoma viral oncogene homologue, LADD: live, attenuated, double-deleted Listeria monocytogenes, SoC: standard of care.

Alternatively, a similar number of studies reported clinical success. Interestingly, each of these studies utilized the GVAX vaccine. The studies in question examined adjuvant GVAX compared to neoadjuvant + adjuvant GVAX (Table 4; NCT00389610 and NCT00084383) [94,95], GVAX + CRS-207 compared to GVAX alone (Table 4; NCT01417000 [96], and GVAX monotherapy compared to GVAX + ipilimumab (Table 3; NCT00836407) [90]. In each study, GVAX was well tolerated, and there was a significant benefit to the median OS and disease-free survival (DFS) in the patient groups receiving neoadjuvant + adjuvant GVAX, GVAX + CRS-207, and GVAX + ipilimumab compared to adjuvant GVAX or GVAX monotherapy, respectively. At the current time, there are no data pertaining to the efficacy of GVAX or other TAVs in treating pancreatic cancer compared to the current standard of care or other non-first-line agents. However, the presence of significant survival benefits observed in patients receiving multiple rounds of GVAX or GVAX in combination with additional agents is promising for future Phase III clinical trials. 

Although less studied in comparison to GVAX, utilization of T-helper type 1 (Th-1) DC vaccines has recently shown promise. The FDA granted a fast-track designation to DOC1021, a Th-1 DC vaccine, in July 2024 for investigation in PDAC following a Phase I trial with early clinical significance. According to the manufacturers, who presented their results at the 2024 American Association for Cancer Research, 12 out of 16 patients remained alive with no significant side effects at 12.9 months following administration. A Phase I clinical trial in PDAC is currently recruiting (Table 1; NCT04157127). The positive results of these studies indicate the potential for success with a variety of TAV modalities, and the field continues to innovate. 

As the newest immunotherapeutic approach being tested in PDAC, cellular therapy development in PDAC has been multifold, with manipulation to target tumor antigens directly or to dismantle the surrounding tumor microenvironment to facilitate synergistic antitumor activity in conjunction with radiation, vaccines, or chemotherapeutics [103]. Examples of antigens comprising the tumor microenvironment that are active areas of investigation include mesothelin and Claudin18.2. In a Phase I trial, CAR-T cells specific for mesothelin were introduced into six patients with metastatic PDAC. Notably, this method was designed to confer an immune response while minimizing off-target toxicity. Although there were no reported dose-limiting toxicities and antitumor activity suggested by a response in liver metastasis in one patient, the primary pancreatic lesion was unaffected, and no CART-mesothelin cells were found on biopsy three to seven days following administration. The best results were stable disease in two patients, and the study suggests a trade-off for an improved safety profile at the expense of transient activity (Table 5; NCT01897415) [104]. Claudin18.2, a tight-junction protein with high levels of expression in gastric cancer and PDAC, presents a structural target for cellular therapy that has been successful in gastric and GEA [105]. However, results when studied in patients with PDAC were lackluster, with only one out of five patients achieving a partial response (Table 5; NCT03159819) [106]. Additional investigations involving both mesothelin and claudin are underway (Table 1; NCT03323944, NCT04581473).

**Table 5:** Completed Cellular Therapy–Based Trials in PDAC. * = data acquired from ASCO Publications; ^+^ = data acquired from PubMed or peer-reviewed publications; ^#^ = data acquired from ClinicalTrials.gov. The confidence interval of 95% is included in parentheses when available. RR: response rate.

NCT	Phase	Intervention	Study Participant	Study Duration	Clinical Outcomes
NCT00965718 ^#,+^, [107]	II	Single Arm: Activated cytokine-induced killer cells	*n* = 20, gemcitabine refractory advanced PDAC	2009–2010	DCR: 25% (3.78 to 46.22) OS: 6.6 weeks (8.6 to 44.6) PFS: 11 weeks (8.8 to 13.2)
NCT01897415 *, [108]	I	Single Arm: Autologous mesothelin-CAR-T cells	*n* = 6 metastatic PDAC	2013–2017	SD: 33.33%
NCT02718859 ^+^, [109]	I, II	Arm A: IRE alone Arm B: IRE + allogenic NK cell therapy	*n* = 40 advanced PDAC	2016–2019	ORR at 2 mo: 63.16% in Arm A vs. 80% in Arm B
NCT03180437 ^+^, [110]	I, II	Arm A1: IRE surgery + single course of γδT cells Arm B: IRE surgery	*n* = 62 locally advanced PDAC	2017–2019	mOS: 14.5 mo in Arm A vs. 11 mo in Arm B. mPFS: 11 mo in Arm A vs. 8.5 mo in Group B
NCT03114631 ^+^, [111]	I, II	Arm A: Autologous DC tumor lysate (5 doses) Arm B: Autologous DC tumor lysate (10 doses) Arm C: Autologous DC tumor lysate (15 doses)	*n* = 26 unresectable PDAC	2017–2019	One-year OS: 78.2% in Arm C vs. 33.8% in control *p* = 0.0001
NCT03159819 *, [106]	I	Single Arm: Autologous CAR-CLD18.2 T cells	*n* = 12, including gastric adenocarcinoma, *n* = 5 advanced or metastatic PDAC patients	2017–2021	ORR: 20%

18-FDG-PET/CT: fludeooxyglucose-18 positron emission tomography/computed tomography, CAR-T: chimeric antigen receptor T-cell, CLD18.2: Claudin 18.2, DC: dendritic cell, IRE: irreversible electroporation, RECIST: response evaluation criteria in solid tumors, RR: response rate, SD: stable disease.

Alternatively, novel approaches to cellular therapy include directly targeting neoplastic antigens expressed on tumor cells or increasing immunogenicity. Aberrant forms of the Mucin family protein (MUC1), a transmembrane glycoprotein expressed on epithelial cell surfaces, acts to increase oncogenic signaling pathways and is an independent prognostic factor in PDAC [112]. Consequently, it has been targeted in multiple Phase I trials in an array of solid tumors, including PDAC (Table 1; NCT05239143). A Phase I trial assessing 26 PDAC patients receiving DC therapy had encouraging early results, with an increase in OS from 33.8% in control to 78.2%, with a dose-dependent relationship evident (Table 5; NCT03114631) [111]. These early results are promising, suggesting that MUC1 may present a target that is reliably expressed in PDAC tumors. 

A large Phase II trial investigating the use of modified autologous T cells in advanced malignancies, including PDAC, is ongoing, involving T cells engineered to express TCRs specific for mutated KRAS G12D in patients with the human leukocyte antigen C 08:02 haplotype (HLA-C*08:02) (Table 1; NCT03935893). A case report by Leider et al. detailed a patient with progressive metastatic PDAC who received a single infusion of genetically engineered T-cell expression of two HLA-C*08:02-restricted TCRs targeting mutant KRAS G12D. This patient experienced a substantial reduction in metastatic lesions, achieving an overall partial response of 72% [113]. The infused T cells continued to proliferate, constituting approximately 2.4% of all T cells in circulation six months post-infusion. Unfortunately, in a subsequent attempt with a different patient, similar therapy failed to produce a meaningful clinical response, and the patient developed progressive disease and passed away within six months [113]. Although these isolated findings suggest the potential of TCR-engineered T cells targeting KRAS G12D mutations to mediate tumor regression in PDAC, the lack of reproducibility underscores the need for further trials to identify reliable indicators and optimize patient selection for this type of cellular therapy.

The use of bispecific antibody-armed activated T cells (BATs) enables the recognition of multiple antigens [114]. Multiple ongoing trials are currently investigating the use of BATs against both EGFR, priming the host immune system to specifically target tumorigenesis and migration, and CD3, promoting auto-apoptosis (Table 1; NCT04137536, NCT03269526). A pair of tandem trials involving anti-CD3 x anti-EGFR BATs is investigating response with different dosing schedules and provoked two instances of stable disease and two instances of complete response following restarting chemotherapy, with an OS of 31 months in 7 total patients (NCT02620865, NCT01420874). Although these completed studies did not meet our inclusion criteria due to low pancreatic cancer patient participant count, this early response is encouraging and suggests a potential role for BATs in mitigating antigen escape and increasing the efficacy of cellular therapy in PDAC.

Finally, cellular therapy delivered with alternative therapies provides a multimodal approach to tumor destruction. In locally advanced pancreatic cancer, irreversible electroporation (IRE) offers a therapeutic non-thermal ablative method that uses electric pulses to create nanopores in the tumor cell membrane, increasing cell instability and rendering it more susceptible to cell death [115]. Researchers have investigated multiple forms of cellular therapy, including allogenic NK cells (Table 5; NCT02718859) [109] and allogenic γδ T cells (Table 5; NCT03180437) [110], with the hope that the IRE would not only induce apoptosis but also enable further penetration into the tumor cell of a boosted immune system [110]. Both trials resulted in promising results in both immunogenicity and clinical response. In combination with NK cells, cancer antigen 19-9 (CA19-9) and carbohydrate antigen (CA242) levels dropped in the experimental arm, with improved quality of life [110]. The combined use of γδ T cells and IRE was similarly promising, with an increase in median OS from diagnosis from 19 months to 22.5 months and an increase in progression-free survival from diagnosis from 15.5 months to 18.5 months compared to IRE alone (*p* = 0.03) [110]. Furthermore, there was a dose-dependent relationship with the cellular therapy for OS, with no increase in adverse events with the addition of the allogeneic cellular therapy. 

## 8. Conclusions and Future Perspectives

Overall, immunomodulatory antibodies have been unsuccessful, and bispecific antibody-based strategies or combined with vaccine therapies may provide methods to counteract tumor-induced immune suppression alongside continued investigation of immunomodulatory antibodies in combination with chemotherapy. In contrast, vaccine and cellular-based therapies have been more recently adapted and have shown preliminary promise as potent approaches to treatment. Regarding cellular therapy, there is a significant need to conduct more Stage II and III trials with larger sample sizes to estimate the broader impact more appropriately. 

This review has some limitations: the clinical trials collection spans over a decade, and the advances in immunotherapy and PDAC-specific approaches advanced significantly during this period. As a result, older trials lack these newer advances and more often present with worse outcomes. Additionally, most of the trials discussed were conducted in Western countries, limiting the applicability to more diverse populations. Future research in larger, more diverse cohorts alongside continued innovation will bolster current evidence toward immunotherapeutic usage to improve patient outcomes by addressing the specific obstacles that pancreatic cancer patients face.

Among 168 initially identified clinical trials, 97 met this review’s inclusion and exclusion criteria. Of these, 33 completed trials (15 antibody therapies, 8 vaccine therapies, 4 antibody + vaccine therapies, and 6 cellular therapies) were recorded for patient clinical outcomes, and 64 ongoing trials continue investigating new strategies for patients with pancreatic cancer. 

## Data Availability

The data that support the findings of this study are available from the corresponding author, upon request.
